# Efficacy of flukicides against *Fasciola hepatica* and first report of triclabendazole resistance on German sheep farms

**DOI:** 10.1016/j.ijpddr.2023.11.001

**Published:** 2023-11-21

**Authors:** Alexandra Kahl, Georg von Samson-Himmelstjerna, Christina Helm, Jane Hodgkinson, Diana Williams, Wiebke Weiher, Werner Terhalle, Stephan Steuber, Martin Ganter, Jürgen Krücken

**Affiliations:** a Institute for Parasitology and Tropical Veterinary Medicine, Freie Universität Berlin, Robert-von-Ostertag-Str. 7, 14163, Berlin, Germany; b Institute of Infection, Veterinary and Ecological Sciences, University of Liverpool, L3 5RF, Liverpool, UK; c Federal Office of Consumer Protection and Food Safety (BVL), Gerichtstraße 49, 13347, Berlin, Germany; d Clinic for Swine and Small Ruminants, Forensic Medicine and Ambulatory Service, University of Veterinary Medicine Hannover, Foundation, Bischofsholer Damm 15, 30173, Hannover, Germany

**Keywords:** *Fasciola hepatica*, Anthelmintic resistance, Triclabendazole, FECRT, Coproantigen ELISA

## Abstract

*Fasciola hepatica* infections lead to severe health problems and production losses in sheep farming, if not treated effectively. Triclabendazole has been used extensively over decades due to its unique efficacy range against all definitive hostfluke stages but published data about the susceptibility of *F. hepatica* to anthelmintics in Germany are lacking. This study aimed to identify current *F. hepatica* infections in German sheep flocks by coproscopic examinations and to evaluate the efficacy of anthelmintics with a focus on triclabendazole in a field study conducted from 2020 to 2022. Initial screening included 71 sheep farms, many of them with known history of fasciolosis. In this highly biased sample set, the frequency of *F. hepatica* infection at individual sheep and farm level were 12.8% and 35.2%, respectively. Additionally, eggs of Paramphistominae were found at frequencies of 4.8% and 15.5% at individual sheep and farm level, respectively. Due to low egg shedding intensity, faecal egg count reduction (FECR) tests could only be conducted on a few farms. The efficacy of triclabendazole was tested on 11 farms and albendazole on one farm, including 3–53 sheep/farm. Individual faecal samples were collected before and two weeks after treatment to evaluate the FECR using the sedimentation or FLUKEFINDER® or a modified FLUKEFINDER® method. On all farms a coproantigen reduction test was conducted in parallel. Lacking efficacy of triclabendazole even at double dosage was shown on one farm associated with a high number of animal losses due to acute fasciolosis. On this farm, the *Fasciola* miracidium development test was additionally performed, revealing a high *in vitro* ovicidal activity of albendazole while closantel was effective *in vivo*. On all other farms, sufficient efficacy of triclabendazole was observed. In conclusion, triclabendazole resistance appears not to be widespread on German sheep farms but, when present, can have serious effects on animal health.

## Graphical abstract

**Figure:**
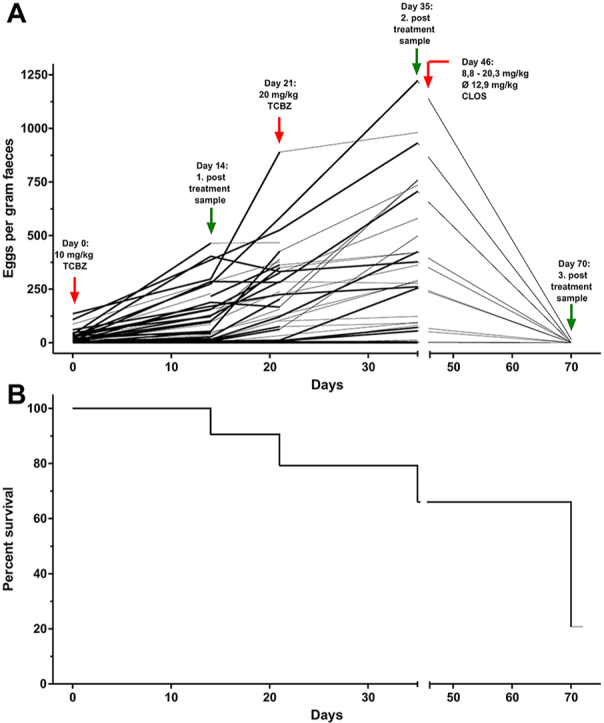

## 1 Introduction

The common liver fluke *Fasciola hepatica* is a trematode parasitising the liver of mammals. The parasite is distributed worldwide (Rojo-Vázquez et al., 2012; Howell and Williams, 2020; Fairweather et al., 2020) and affects in particular ruminants, but also a large range of different other hosts (Beesley et al., 2018; Mas-Coma et al., 2019), including humans (Saba et al., 2004). Lymnaeid snails serve as intermediate hosts for the diheteroxenic life cycle of the parasite (Beesley et al., 2018). *Galba truncatula* is the most important snail species being involved in the transmission of *F. hepatica* in Europe (Charlier et al., 2014). Since the intermediate host is dependent on moist environmental conditions, *F. hepatica* typically occurs in humid regions. Liver fluke infections are becoming increasingly common in ruminants (Skuce and Zadoks, 2013; Beesley et al., 2018; John et al., 2019) and may lead to considerable economic losses in sheep and cattle (Schweizer et al., 2005; Rojo-Vázquez et al., 2012; Gordon et al., 2012; Charlier et al., 2014; Beesley et al., 2018) and is a severe, potentially life-threatening health problem particularly in sheep (Skuce and Zadoks, 2013; Pérez-Caballero et al., 2018). Traditionally, there are three courses of the disease to be distinguished: acute, subacute and chronic clinical manifestations (Rojo-Vázquez et al., 2012), based on the quantity of metacercariae ingested by the host animal (Fiss et al., 2013) and its genetic susceptibility. In sheep, clinical signs extend from chronic wasting associated with emaciation, oedema and anaemia (Sargison and Scott, 2011) to sudden deaths in acute cases (Sargison and Scott, 2011; Rojo-Vázquez et al., 2012; Fiss et al., 2013; Skuce and Zadoks, 2013; Forbes, 2017).

For the treatment of *F. hepatica* infections there are several anthelmintics from different drug classes available. The most commonly used anthelmintic is triclabendazole (TCBZ) (Walker et al., 2004; Halferty et al., 2009) since it is the only compound effective against mature and juvenile mammalian stages of the parasite (Fairweather, 2011a; Fairweather et al., 2012; Skuce and Zadoks, 2013). Other established flukicides, including albendazole (ABZ), closantel (CLOS), nitroxynil, clorsulon, oxyclozanide and rafoxanide only demonstrate high efficacy against the mature parasites (Fairweather, 2011a), making TCBZ the drug of choice to control acute *F. hepatica* infections (Brockwell et al., 2014). The unique efficacy of TCBZ against all fluke stages in the host resulted in an over-reliance on this substance and the development of resistance (Fairweather et al., 2020). The first incidence of TCBZ resistance was reported in 1995 in Australia (Overend and Bowen, 1995). During recent years, there have been numerous reports about lack of TCBZ efficacy against *F. hepatica* in ruminants worldwide and Flanagan et al. (2011b) described resistance to TCBZ as a spreading but under-recognised problem. In Europe, lack of efficacy of TCBZ in ruminants has been reported in Wales (Thomas et al., 2000; Daniel et al., 2012; Kamaludeen et al., 2019), England (Kamaludeen et al., 2019); Scotland (Mitchell et al., 1998; Daniel et al., 2012; Gordon et al., 2012), Ireland (Mooney et al., 2009; Hanna et al., 2015), the Netherlands (Moll et al., 2000; Gaasenbeek et al., 2001) and Spain (Alvarez-Sánchez et al., 2006). Further reports originate from Australia (Brockwell et al., 2014; Elliott et al., 2015), Argentina (Olaechea et al., 2011; Larroza et al., 2023) and Peru (Ortiz et al., 2013).

Different diagnostic methods can be applied for the evaluation of anthelmintic efficacy; however, no standard protocol has been established for *F. hepatica* (Fairweather, 2011a; Fairweather et al., 2012; Brockwell et al., 2014; Novobilský and Höglund, 2015; Solana et al., 2016). The most frequently practiced method to determine drug efficacy is the faecal egg count reduction test (FECRT), with anthelmintic treatment being defined effective if a 95% faecal egg count (FEC) reduction is ascertained on day 14 (or 21) post-treatment (Daniel et al., 2012; Fairweather, 2011a; Fairweather et al., 2012; Solana et al., 2016). However, the exclusive implementation of the FECRT for drug efficacy analysis against *F. hepatica* has practical limitations: *Fasciola hepatica* shows a long prepatent period ranging from 8 to 12 weeks (Calvani et al., 2018; Mezo et al., 2022), so that eggs can only be coproscopically detected at a later stage of the infection, making the FECRT not applicable for animals in the prepatent stage (Almazán et al., 2001; Duthaler et al., 2010; Fairweather, 2011b; Skuce and Zadoks, 2013; Mazeri et al., 2016; Mezo et al., 2022). Moreover, *F. hepatica* egg shedding is intermittent (Gordon et al., 2012; Kelley et al., 2021), and FEC can potentially be false-negative and highly variable in a single animal over time. In addition, TCBZ is the only currently available drug effective against juvenile flukes, thus, the efficacy of adulticidal drugs is more difficult to determine, as juvenile or immature flukes are not effectively treated and may mature within the two weeks until the post-treatment sample is collected (Coles et al., 2006; Novobilský et al., 2016). Furthermore, coproscopic methods may show false-positive results following effective treatment and elimination of the parasites, as fluke eggs are stored in the gallbladder and can be shed in the faeces for weeks even if the flukes have been successfully killed by the anthelmintic (Chowaniec and Darski, 1970; Fairweather, 2011a).

Hence, it is best practice to combine the FECRT with another diagnostic method to evaluate anthelmintic efficacy reliably (Fairweather, 2011b; Hanna et al., 2015) such as the coproantigen reduction test (CRT), which has been proven to show adequate results for the diagnosis of anthelmintic efficacy in *F. hepatica* in the field (Flanagan et al., 2011a, 2011b; Gordon et al., 2012; Brockwell et al., 2013; Hanna et al., 2015). *Fasciola hepatica* releases antigens that are excreted with the faeces of its host (Almazán et al., 2001) and a coproantigen-ELISA (cELISA) is able to detect infections with a single fluke in sheep (Mezo et al., 2004). In contrast to FECs, a positive cELISA proves the presence of metabolically active flukes (Martínez-Pérez et al., 2012). Moreover, various studies reported that fluke antigens can be detected earlier than eggs (Almazán et al., 2001; Flanagan et al., 2011a; Flanagan et al., 2011b; Valero et al., 2009). In contrast, Gordon et al. (2012) did not observe that coproantigens can be detected before parasite eggs are shed during their field study, but overall, the authors still evaluated the cELISA as suitable for efficacy tests and more convenient than FECs.

Another method to evaluate anthelmintic efficacy is the *Fasciola* miracidium development (MDT) or egg hatch test (FEHT), a low-cost *in vitro* method, which is able to distinguish between ABZ-susceptible and ABZ-resistant *F. hepatica* isolates by incubating the isolated *F. hepatica* eggs in ABZ solutions of different concentrations and evaluating the development and hatching rates of the treated eggs compared to untreated control eggs (Alvarez et al., 2009; Canevari et al., 2014; Robles-Pérez et al., 2014; Novobilský et al., 2016; Ceballos et al., 2019). According to Alvarez et al. (2009), this test does not work for TCBZ. Reasons for this might be the highly lipophilic nature of TCBZ or the high binding affinity for different proteins, both impeding the penetration of the drug through the eggshell, or a non-microtubule related mode of action of TCBZ, different from the mode of action of other benzimidazoles (Alvarez et al., 2009).

Until now, no published data are available regarding the susceptibility of *F. hepatica* to flukicides in Germany. Therefore, the primary aim of this study was to investigate the efficacy of TCBZ in German sheep flocks. Unexpectedly, the number of sheep flocks with sufficient prevalence and egg shedding intensity to conduct a FECRT was very low during the evaluation period (most likely due to the extremely dry weather conditions in 2020 in Germany). Therefore, many sheep flocks were screened and data on frequency and intensity of egg shedding are also reported.

## 2 Materials and methods

### 2.1 Investigation on occurrence of *F. hepatica* on German sheep farms

To identify sheep flocks suitable to conduct a FECRT, data about the current occurrence of *F. hepatica* on German sheep farms were collected. In total, 1673 individual faecal samples from 71 German sheep farms located in different regions of the country were coproscopically examined for the presence of *F. hepatica* eggs between December 2020 and August 2022. At the beginning of the study, the authors focussed on the examinations of farms located in Lower Saxony due to the collaboration with the University of Veterinary Medicine in Hannover and a high number of *F. hepatica* findings in the past in this federal state. Since the frequency of *F. hepatica* findings turned out to be lower than expected and a sufficient number of infected flocks was not detected in Lower Saxony during the winter season 2020/2021, the geographic range for coproscopic examinations was expanded from the beginning of 2021 on and eligible farms in other regions of the country were examined. Farms were specifically selected due to previous *F. hepatica* infections (as recorded by the Clinic for Swine and Small Ruminants in Hannover or by local veterinarians). Moreover, farms with a high probability of *F. hepatica* occurrence due to wet pasture conditions were contacted by systematically calling sheep farms in the coast regions in Lower Saxony and Schleswig-Holstein. After identifying three highly infected sheep farms in the region of Paderborn (North Rhine Westphalia), a local veterinarian collected faecal samples from several more farms in this region and sent them to Berlin for coproscopic examinations. In addition, the objectives of the study were published in three national veterinary and sheep farmer journals from 2021 to 2022 addressing the target groups of sheep farmers and veterinarians and asking to contact the authors if sheep farms with anamnestic *F. hepatica* infections or a high probability of *F. hepatica* occurrence are known.

Individual faecal samples were either submitted by farmers by post or the farms were visited by the authors to collect individual samples personally. The number of examined individual samples ranged from 3 to 79 samples per farm. Farmers were asked to specifically select individual animals with a poor body condition, oedema, or poor wool quality for sampling since poor body conditions can result from chronic fasciolosis (Kahl et al., 2021).

The coproscopic examination was offered free of charge for the farmers. Inclusion criteria were access to a pasture on which the occurrence of *F. hepatica* is likely due to moist conditions and/or previous *F. hepatica* infections on the farm and a farm location in Germany.

Each faecal sample was individually analysed using either a standard sedimentation method, the FLUKEFINDER® method or a combination of both methods (“Modified FLUKEFINDER®“) (Kahl et al., 2023). Trematode eggs were counted separately for *F. hepatica* and Paramphistominae (rumen flukes) which were distinguished according to their different colours (Mazeri et al., 2016).

### 2.2 Investigation of flukicide resistance

The field trial to evaluate anthelmintic efficacy in German sheep flocks was conducted from February 2021 to June 2022. In total, 12 sheep farms from different federal states of Germany were included in the trial. Three of the farms were located in Lower Saxony, three in North Rhine-Westphalia, two in Brandenburg, two in Schleswig-Holstein, one in Mecklenburg-Western Pomerania and one in Baden-Wuerttemberg. The number of treated animals with available samples before and 14 days after treatment varied from 9 to 71 sheep, including sheep that turned out to have a FEC of zero in the first faecal examination. The number of sheep coproscopically *F. hepatica*-positive before treatment varied from 3 to 35 per farm. In 11 out of 12 flocks, the efficacy of TCBZ was evaluated. However, since TCBZ is non-licensed for dairy livestock, ABZ was tested on the only included dairy sheep farm instead. All farms were visited at least twice. Sheep were individually treated with the anthelmintic according to the individual bodyweight (bw) and rectally sampled on day 0. On day 14 post treatment, the farms were revisited for the collection of the post treatment samples to evaluate the treatment success. On one farm, the collection of the post-treatment samples was performed on day 15 post treatment. A two-week-interval between the collection of the pre- and post-treatment samples was described in the literature for the FECRT (Mooney et al., 2009; Fairweather 2011a; Fairweather et al., 2012; Solana et al., 2016) and the CRT (Fairweather, 2011a; Flanagan et al., 2011b; Brockwell et al., 2014) and Flanagan et al. (2011a) assessed day 14 post-treatment as a robust re-sampling time when combining FECRT and CRT.

The collection faecal samples and oral treatment were approved by each visited federal state of Germany, confirmed in written form, that rectal sampling and oral treatment were not considered to be an animal experiment within the framework of the German law (Tierschutzgesetz) and the EU directive 2010/63/EU.

#### 2.2.1 Clinical examination, weighing, treatment, sampling

On day 0, sheep were individually weighed on a mobile animal balance (resolution: 0.1 kg) and orally treated with a licensed TCBZ preparation (ENDOFLUKE® 100 mg/ml, Livisto aniMedica GmbH, Senden-Boesensell, Germany or Cydectin® TriclaMox 1 mg/ml + 50 mg/ml, Zoetis Deutschland GmbH, Berlin, Germany) in the dosage of 10 mg TCBZ/kg body weight (bw) or ABZ preparation (Valbazen® 1,9%, Elanco Deutschland GmbH, Bad Homburg, Germany) in the dosage of 7.5 mg ABZ/kg bw. A disposable syringe (size: 5 ml, 10 ml, or 20 ml) was used to ensure dosing exactly adjusted to the individual bodyweight. Finally, a faecal sample was taken rectally from each individual animal. On day 14 (day 15 on one farm) post treatment, each individual sheep was re-sampled.

A second set of post-treatment samples was collected on one farm on day 21 and on another farm on day 28, since *F. hepatica* eggs were still present in the first post-treatment samples (14/15 days post treatment).

On one farm, on which TCBZ did not lead to a FECR nor to a decrease of coproantigen levels at day 14 post treatment, a second oral treatment of the study population with the double dose of TCBZ (20 mg/kg bw) was conducted on day 21 post first treatment and further post treatment samples were collected on day 35 (14 days after the second treatment).

Faecal samples were stored in a transportable electric cool box and transferred to the laboratory in Berlin within one to two days.

#### 2.2.2 Faecal egg count reduction test (FECRT)

The FECRT was performed on a farm level using paired data pre- and post-treatment for individual animals. After arrival in the laboratory, samples were stored at 4 °C until coproscopic examinations within at least seven days after sample collection.

In contrast to the coproscopic examinations conducted for screening sheep flocks for the presence of *F. hepatica* eggs, the FECRT was performed using only two different sedimentation techniques (“Modified FLUKEFINDER®” and FLUKEFINDER®). The standard sedimentation method was not applied for the FECRT. The pre- and post-treatment examinations of the first three farms included were conducted using a method combining a standard sedimentation technique with the FLUKEFINDER® method (Soda Springs, Idaho, USA) using 10 g of faeces per sample as described recently (“Modified Flukefinder”) (Kahl et al., 2023). However, since the collection of 10 g of faeces turned out to be a practical constraint, the standard protocol of the FLUKEFINDER® using only 2 g of faeces was applied on farms 4–12 as described in Kahl et al. (2023).

#### 2.2.3 Coproantigen reduction test (CRT)

The CRT was also performed at the individual animal level. After arrival in the laboratory, samples were either stored at 4 °C or frozen at −20 °C until examination (maximum storage time at 4 °C or −20 °C: 3 months) as recommended by Flanagan et al. (2011a).

For the CRT, a commercially available cELISA kit (BIO K 201/2 – Monoscreen AgELISA Fasciola hepatica, Bio-X Diagnostics S.A., 5580 Rochefort, Belgium, batches FASA20L03, FASA21M11, FASA21L23) was used according to the manufacturer's instructions. Absorbances were read at 450 nm using an Epoch microplate spectrophotometer (Bio Tek Instruments, Winooski, Vermont, USA). After subtracting the OD of the negative control from sample and positive control Ods, a relative OD was calculated as: (Delta OD Sample * 100)/Delta OD positive control = relative OD (%)

According to the manufacturer's instructions, the sample's status (positive or negative for *F. hepatica*) was determined using a lot-specific threshold value, which was 8.0% relative OD. However, during the course of the study, several coproscopically positive pre-treatment samples with a calculated relative OD value of less than 8.0% were found. Hence, it was decided to decrease the threshold value for this study to 2.0% of the positive control OD value for all samples to avoid false negative assertions referring to the cELISA. At 2% relative optical density, all FEC positive samples were also positive in cELISA.

#### 2.2.4 *Fasciola* miracidium development test (FMDT)

The FMDT was performed with the eggs collected on one farm, on which TCBZ failure was observed. This was done to evaluate, whether ABZ was still effective against this field population.

The protocol from Alvarez et al. (2020) was implemented with slight modifications. For the assay, *F. hepatica* eggs were isolated from faecal samples: The sediments from positive individual samples were pooled after coproscopic examination as part of the FECRT. The pooled samples were further cleaned by another round of purification on the FLUKEFINDER® followed by numerous 3-min sedimentation cycles with tap water in a 250 ml beaker until the sediment was macroscopically as clean as possible. The sediments were transferred into a 50 ml centrifugation tube. An egg suspension of 200 eggs/ml was prepared using 10 mM sodium phosphate buffer (pH 7) (NaPi) as a solvent instead of tap water as indicated in the protocol from Alvarez et al. (2020). The suspension was subdivided and transferred into twenty 7 ml-cell culture tubes (Nunc™ Thermo Fisher Scientific™, Waltham, Massachusetts, USA) with 1 ml egg suspension per tube. A 5 mM ABZ stock solution was prepared (13.26 mg ABZ dissolved in 10 ml methanol acidified with 20 μl HCl (37%)). Three different ABZ working solutions (500 μM; 50 μM and 5 μM) were generated originating from the 5 mM stock solution. The ABZ efficacy was tested in three different final concentrations (5 μM, 0.5 μM and 0.05 μM) by adding 10 μl of the different working solutions to the respective tubes. Each concentration was assessed using five replicates. Untreated eggs (5 replicates) exposed only to 1% methanol were included as negative control. The tubes were incubated in the dark at 25 °C for 12 h and subsequently washed three times with 10 mM NaPi to remove the drug. Afterwards, the washed eggs were incubated in 1 ml NaPi in the dark at 25 °C for 15 days. On day 16, eggs were exposed to daylight for 4 h before adding 10 μl 10% buffered formalin (pH 7.8) to each egg suspension to stop the development. The eggs were carefully inspected using a microscope (400 × magnification) to identify completely undeveloped eggs and discriminate them from partially developed eggs and egg shells after hatching of miracidia. Fully embryonated eggs and empty eggshells after hatching of miracidia were counted together as “developed eggs”. The percentage of developed eggs was calculated for each replicate in each ABZ concentration and the negative controls. The egg development rate in the negative controls in each assay was required to be at least 70% to consider the assay as valid. The ABZ ovicidal activity was calculated by means of the following formula:

Ovicidal activity = [(% eggs developed in control - % eggs developed after drug incubation)/% eggs developed in control] x 100.

### 2.3 Statistical analyses

The statistical analyses were performed using R version 4.2.0 in R Studio version 2022.07.1.

The results of the faecal examinations regarding the occurrence of *F. hepatica* and Paramphistominae on German sheep farms were grouped according to the time points of the sampling: December 2020 (start of the examinations)-March 2021; April 2021–September 2021; October 2021–March 2022; April 2022–August 2022 (end of the examinations). The visualisation of the occurrence of the parasites on a map was performed using Microsoft Excel 2019 with Microsoft 3D Maps (Microsoft Corporation, Redmond, WA, USA).

The 95% confidence intervals (CI) for the occurrence frequencies of *F. hepatica* and Paramphistominae were calculated using the binom. wilson function in the R package epitools (version 0.5–10.1). Tests for statistical significance of the differences between *F. hepatica* and Paramphistominae occurrences were performed using the tab2by2. test in the R package epitools. Odds ratios were calculated using the mid-p exact method implemented in the or. midp command from the same package.

The reduction in egg excretion after anthelmintic treatment in the efficacy trial was calculated with 95% credibility intervals (the Bayesian equivalent to confidence intervals) using the R package eggCounts version 2.3–2 (Wang et al. 2017, 2018), which implements Bayesian hierarchical models to assess the efficacy of treatment (using the model common efficacy on herd level without zero-inflation).

The calculation of the net optical densities in the cELISA and the final percentual result using the formula given by the manufacturer was performed using Michiels et al., 1987 (Microsoft Corporation, Redmond, WA, USA).

Cohen's kappa coefficients were calculated using the CohenKappa function from the DescTool package (version 0.99.48) to measure the level of agreement between the results detected by the FLUKEFINDER®method and the cELISA (farms no. 3–12) depending on the threshold value used for assessing the cELISA result. Cohen's kappa values were calculated independently for cELISA results with thresholds set to 2.0% and 8.0%, respectively. The results of both calculation approaches were compared using the table by Landis and Koch (1977).

For visualisation of the results in graphs, Graph Pad Prism 5.03 (GraphPad Software, San Diego, CA, USA) was used.

## 3 Results

In total, 71 farms were included in the initial screening. Supplemental Table S1 provides information about the locations, the time of examination, the FEC method applied on each respective farm and the FEC results of all farms included in the study.

### 3.1 Occurrence of *Fasciola hepatica* and Paramphistominae on German sheep farms

**Fig. 1:**
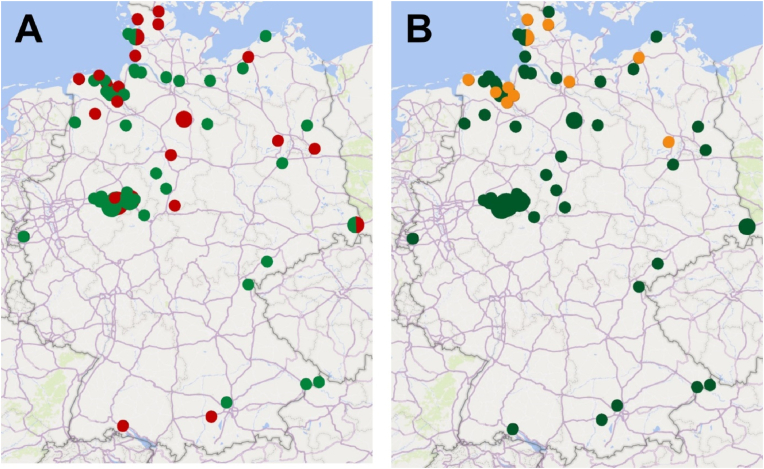
Occurrence of *F. hepatica* (A) and Paramphistominae (B) on German sheep farms from 2020 to 2022 using coproscopic methods. Individual farm locations were defined by the combination of the village name and the ZIP code. If two farms were located in the same village, they are represented by a single, larger circle. Locations with detection of *F. hepatica* are marked in red on map A, locations with detection of Paramphistominae are marked in orange on map B and locations where trematodes were not detected, are marked in green. Red-green coloured dots on map A show the occurrence of *F. hepatica*-positive and *F. hepatica*-negative farms in the same location. Orange-green coloured dots on map B show the occurrence of Paramphistominae-positive and Paramphistominae-negative farms in the same location.

The results of the investigation of the current occurrence of *F. hepatica* on German sheep farms are summarised in Table 1 and visualised in Fig. 1. Out of the 1673 individual sheep investigated during the study, 214 sheep (12.8%, 95% CI 11.3–14.5%) on 25 out of 71 farms (35.2%, 95% CI 25.1–46.8%) were shedding *F. hepatica* eggs in their faeces. The frequency at which eggs of Paramphistominae were found was lower: only 81 individual animals (4.8%, 95% CI 3.9–6.0%) on 11 out of 71 farms (15.5%, 95% CI 8.9–25.7%) were coproscopically positive for Paramphistominae, resulting in a significantly lower occurrence of Paramphistominae on farm level as well as on individual animal level (p = 0.007 and p < 0.001, respectively) (Table 1). In addition, there were considerable differences between seasons. *Fasciola hepatica* was found significantly more often than rumen flukes in winter 2021/22 (OR 1.8) and winter 20/21 (OR 4.4), while in summer 2021 Paramphistominae were found significantly more often than *F. hepatica* (OR 0.5). Co-infections with both groups of trematodes were observed on 8 of 71 farms (11.3%, 95% CI 5.8–20.7%) and in 21 of 1673 individual sheep (1.3%, 95% CI 0.8–1.9%). The odds to find Paramphistominae on farms that were positive for *F. hepatica* was 3.6-fold higher than on *F. hepatica* negative farms but this difference was not significant (p = 0.070, mid-p exact test).

**Table 1:** Comparisons between frequency of infection with *Fasciola hepatica* and Paramphistominae and between seasons in coproscopic data before treatment.

		*F. hepatica*		Paramphistominae			
	N^a^	n^b^	Frequency % (95% CI)	n^b^	Frequency % (95% CI)	P value^c^	OR^c^ (95% CI)
**Comparisons between trematode groups**							
**Farm level**							
Winter 20/21^d^	12	5	44.67 (19.3–68.0)	3	25.0 (8.9–53.2)	0.430	2.0 (0.35–13.9)
Summer 21	10	6	60.0 (31.3–83.2)	3	30.0 (10.8–60.3)	0.220	3.2 (0.5–24.8)
Winter 21/22	39	12	30.8 (18.6–46.4)	2	5.1 (1.4–16.9)	0.003	7.6 (1.8–56.6)
Summer 22	10	2	20.0 (5.7–51.0)	3	20 (5.7–51.0)	0.652	0.6 (0.1–5.2)
Total winter	51	17	33.3 (22.0–47.0)	5	33.3 (22.0–47.0)	0.004	4.5 (1.6–15.0)
Total summer	20	8	40.0 (21.9–61.3)	6	30.0 (14.5–51.9)	0.531	1.5 (0.4–6.0)
Total	71	25	35.2 (25.1–46.8)	11	15.5 (8.9–25.7)	0.007	2.9 (1.3–6.8)
**Individual level**							
Winter 20/21	248	50	20.2 (15.6–25.6)	30	12.1 (8.6–16.7)	0.015	1.8 (1.1–3.0)
Summer 21	411	16	3.9 (2.4–6.2)	32	7.8 (5.6–10.8)	0.018	0.5 (0.3–0.9)
Winter 21/22	792	134	16.9 (14.5–19.7)	8	1.0 (0.5–2.0)	<0.001	4.4 (3.0–6.4)
Summer 22	222	14	6.3 (3.8–10.3)	11	5.0 (2.8–8.7)	0.783	1.3 (0.6–3.0)
Total winter	1040	184	17.7 (15.5–20.1)	38	3.7 (2.7–5.0)	<0.001	5.6 (4.0–8.2)
Total summer	633	30	4.7 (3.3–6.7)	43	6.8 (5.1–9.0)	0.119	0.7 (0.4–1.1)
Total	1673	214	12.8 (11.3–14.5)	81	4.8 (3.9–6.0)	<0.001	2.9 (2.2–3.8)
**Comparisons between seasons on individual level**						P value^c^	OR^c^ (95% CI)
***F. hepatica***							
Total winter	1040	184	17.7 (15.5–20.1)			<0.001	4.3 (2.9–6.5)
Total summer	633	30	4.7 (3.3–6.7)				
**Paramphistominae**							
Total winter	1040			38	3.7 (2.7–5.0)	0.004	0.5 (0.3–0.8)
Total summer	633			43	6.8 (5.1–9.0)		

a Total investigated.

b Total positive.

c Odds ratios and p values calculated using the mid-p exact method and *F. hepatica* as reference level.

d Definition of seasons: Winter 20/21: December 2020–March 2021; Summer 21: April 2021–September 2021; Winter 21/22: October 2021–March 2022; Summer 22: April 2022–August 2022.

A seasonal variation in the occurrence of *F. hepatica* was observed: The highest number of patently *F. hepatica* infected sheep was found in the winter seasons with 20.2% and 16.9% of the examined samples containing at least one *F. hepatica* egg in winter 2020/2021 (December 2020–March 2021) and 2021/2022 (October 2021–March 2022), respectively. In contrast, only 3.9% and 6.3% of the examined samples were tested positive for *F. hepatica* during summers 2021 (April 2021–September 2021) and 2022 (April 2022–August 2022), respectively. The difference between the occurrences of *F. hepatica* in the winter seasons and the summer seasons was significant in the mid-p exact test (p < 0.001).

The highest number of animals testing positive for Paramphistominae, in relation to the total number of investigated animals, was found in winter 2020/2021 (12.1%), whereas the percentage of positive samples in the following winter 2021/2022 was much lower (1.0%) and even lower compared to both summers 2021 and 2022 (7.8% and 5.0%, respectively). For rumen flukes, no statistically significant difference was detected between winter and summer (p = 0.823).

The geographical occurrence of *F. hepatica*-positive and *F. hepatica*-negative farms and the occurrence of Paramphistominae-positive and Paramphistominae-negative farms is shown on maps in Fig. 1.

### 3.2 Inter rater agreement between FLUKEFINDER® and coproantigen ELISA

Cohen's kappa coefficients were calculated twice to measure the level of agreement between the coproscopic FLUKEFINDER® method and the copro-immunological method using the results from farms no. 3–12. In total, 612 individual faecal samples (pre- and post-treatment samples) were analysed with both methods in parallel to assess whether an individual animal was positive or negative for a patent *F. hepatica* infection.

When considering only samples with an OD value of ≥8.0% of the positive control OD value (i.e. the lot-specific threshold value) as positive for *F. hepatica*, the calculated kappa coefficient was 0.797 (95% CI: 0.749–0.846). According to the nomenclature of Landis and Koch (1977), this can be described as a “substantial” strength of agreement. Compared to that, when all samples with an OD value of ≥2.0% of the positive control OD value (i.e. the threshold value applied within this study) were considered as positive for *F. hepatica*, the calculated kappa coefficient was 0.805 (95% CI: 0.757–0.852), which is slightly higher than the kappa value in the first calculation, but also a “substantial” agreement according to the nomenclature of Landis and Koch (1977). When looking at the 95% CI values, the increase in Cohen's kappa was not significant.

### 3.3 Efficacy study to evaluate the flukicidal activity of TCBZ and ABZ

**Fig. 2:**
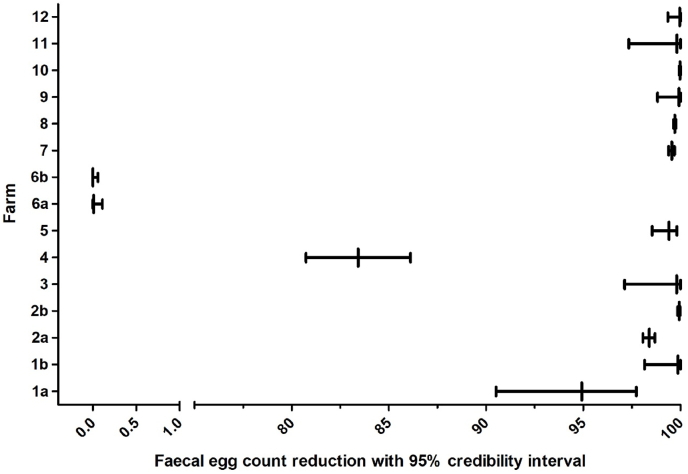
Calculated faecal egg count reduction (FECR) with 95% credibility intervals for *F. hepatica* on all 12 farms. The EggCounts package was used in R to calculate FECR and 95% credibility limits assuming a common efficacy for all animals. Albendazole was used as flukicide on farm 1 while triclabendazole was tested on farms 2–12. Farm 1a: FECR day 0 to day 14 post treatment, farm 1 b: FECR day 0 to day 21 post treatment, farm 2a: FECR day 0 to day 15 post treatment, farm 2 b: FECR day 0 to day 28 post treatment, farm 6a: FECR day 0 to day 14 post treatment, farm 6 b: FECR day 21 to day 35 post treatment.

The results of the field trial are summarised in Table 2 and Fig. 2. Sufficient efficacy of TCBZ as evidenced by negative cELISA (cELISA result below the chosen threshold value of 2.0% relative OD) of all originally positive animals two weeks after treatment was shown on nine out of 11 farms, on which TCBZ efficacy was tested. Sufficient efficacy of TCBZ based on the FECRT results (>95% FECR two weeks after treatment) was also shown on nine out of 11 farms, on which TCBZ efficacy was tested.

**Table 2:** Faecal egg count reduction (FECR) (calculated using the EggCounts package in R Studio, assuming a common drug efficacy for all animals) and coproantigen reduction test (CRT) results for *F. hepatica* on all 12 farms included in the efficacy trial.

Farm	Federal state	Method	Drug	Product	N total	N paired FEC	n	FECR %	2.5% CL	97.5% CL	N paired cELISA	cELISA positive before/after^d^
1a^a^	Lower Saxony	Mod. FLUKEFINDER®	ABZ	Valbazen 1.9%	13	13	4	94.92	90.51	97.73	13	3/1
1 b^b^	Lower Saxony	Mod. FLUKEFINDER®	ABZ	Valbazen 1.9%	13	4	4	99.87	98.15	100	4	3/0
2a^a^	Mecklenburg-Western Pomerania	Mod. FLUKEFINDER®	TCBZ	Cydectin TriclaMox	46	43	35	98.4	98.07	98.69	44	35/1
2 b^b^	Mecklenburg-Western Pomerania	Mod. FLUKEFINDER®	TCBZ	Cydectin TriclaMox	46	41	33	99.94	99.85	99.99	n.d	n.d.
3	Schleswig-Holstein	Mod. FLUKEFINDER®	TCBZ	Cydectin TriclaMox	79	71	3	99.81	97.12	100	4	3/0
4	North Rhine Westphalia	FLUKEFINDER®	TCBZ	Endofluke	55	46	8	83.42	80.73	86.11	49	9/0
5	Brandenburg	FLUKEFINDER®	TCBZ	Endofluke	32	32	12	99.41	98.54	99.82	32	12/0
6a^a^	Lower Saxony	FLUKEFINDER®	TCBZ	Endofluke	53	36	24	0.01	0	0.11	37	30/33
6 b^c^	Lower Saxony	FLUKEFINDER®	TCBZ	Cydectin TriclaMox	53	27	22	0	0	0.06	29	27/27
7	North Rhine-Westphalia	FLUKEFINDER®	TCBZ	Endofluke	48	43	28	99.55	99.39	99.69	45	33/0
8	Baden-Wuerttemberg	FLUKEFINDER®	TCBZ	Endofluke	30	30	30	99.71	99.64	99.77	30	30/0
9	Schleswig-Holstein	FLUKEFINDER®	TCBZ	Cydectin TriclaMox	21	13	7	99.92	98.82	100	13	5/0
10	North Rhine-Westphalia	FLUKEFINDER®	TCBZ	Cydectin TriclaMox	17	17	8	99.98	99.93	100	17	9/0
11	Brandenburg	FLUKEFINDER®	TCBZ	Endofluke	9	9	5	99.82	97.34	100	9	4/0
12	Lower Saxony	FLUKEFINDER®	TCBZ	Endofluke	27	27	13	99.96	99.35	100	27	12/0

N total, total number of coproscopically examined individual animals on the farm before flukicidal treatment; N paired, total number of paired samples pre and post treatment; n, number of coproscopically F. hepatica-positive animals before treatment; CL, credibility limit; ABZ, albendazole; TCBZ, triclabendazole.

a FECR/coproantigen reduction from day 0 to day 14 (1a and 6a)/to day 15 (2a).

b FECR/coproantigen reduction from day 0 to day 21 (1 b)/to day 28 (2 b).

c FECR/coproantigen reduction from day 21 to day 35 after treatment with the double dose of TCBZ on day 21.

d Number of samples positive in the coproantigen ELISA before/after treatment using the 2% of the positive control threshold.

High efficacy of ABZ was proven on one dairy sheep farm (farm no. 1) with negative cELISA results and FECR >95% three weeks after treatment. On this farm, a negative coproscopic as well as a negative cELISA result was observed in 12 out of 13 post-treatment samples from day 14. However, one of the post treatment samples still contained a few parasite eggs in the coproscopic examination (0.8 EPG on day 14 post treatment compared to 8.5 EPG before treatment) and therefore, an egg count reduction of 95% was not obtained on the farm level on day 14. Moreover, the cELISA result (4.7% of positive control) was slightly above the chosen threshold value of 2.0% for the egg-positive sample. Therefore, a second set of post-treatment samples was collected on day 21 from all four individual animals that had originally been positive for *F. hepatica* on day 0. No parasite eggs were found in the samples from day 21, leading to an egg count reduction of 100%. Furthermore, the cELISA result was negative for all individual samples on day 21 post treatment. The *F. hepatica* population on this farm was therefore considered to be ABZ susceptible.

On one out of the 11 TCBZ-treated farms (farm no. 2), the egg count reduction already exceeded 95% on day 15 post treatment (FECR: 98.4%). Nevertheless, a second set of post-treatment samples was collected on day 28 as some parasite eggs were still present in the samples on day 15. On day 28, the FECR was >99.9%. Moreover, one individual animal also showed a cELISA result slightly above the chosen threshold of 2% on day 15 (4.58% of positive control), but a negative coproscopic result at the same time. A second set of post-treatment samples from all animals was collected on this farm on day 28 and the coproscopic result of this individual animal (and all other animals) was negative again. The cELISA was not repeated with the second post-treatment samples, since the authors had not decided to lower the threshold for the cELISA from 8.0% to 2.0% of positive control at this point of the study.

On another one out of the 11 TCBZ-treated farms (farm no. 4), a negative cELISA result on day 14 post treatment was seen in all individual post-treatment samples, but one individual sheep of this flock with the highest pre-treatment egg count (154 EPG) still showed a high number of *F. hepatica* eggs in the sample 14 days post treatment (81 EPG), leading to an egg count reduction of only 83.42 % (95% CL 80.73%–86.11%) on herd level. Unfortunately, it was not possible to obtain further samples from this farm since the farmer did not provide further samples from this animal.

**Fig. 3:**
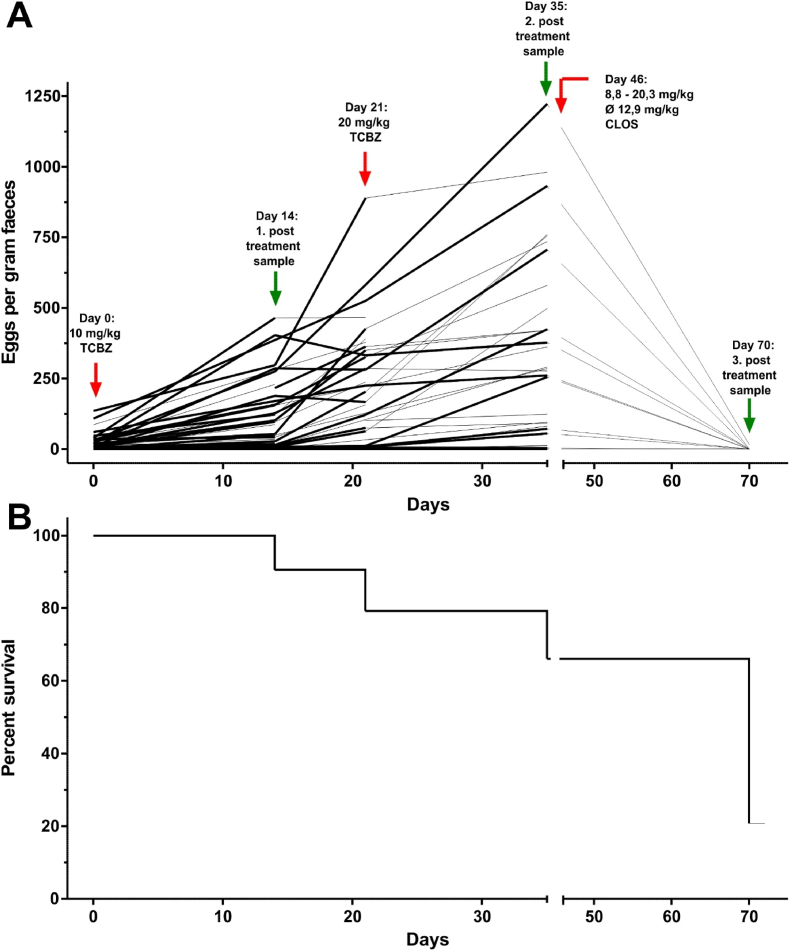
Faecal egg count development of individual sheep (A) and survival (B) shown as Kaplan-Meier plot of the study group on farm no. 6 during the course of the study. The CLOS treatment on day 46 was conducted by the farmer. No faecal samples were available for examination from that particular day.

On another farm (farm no. 6), a high number of sudden deaths was observed daily among recently clinically healthy animals. Some of the deceased animals were pathologically examined and massive infections with juvenile *F. hepatica* stages were observed. On this farm, 53 out of 1300 ewes and lambs of the flock were enrolled in the efficacy trial as a study population and the animals were treated with TCBZ at the recommended dose (10 mg/kg bw) on day 0. The egg counts and coproantigen levels of those 48 animals that were still alive on day 14 post treatment were even higher compared to the pre-treatment results. Hence, a second treatment attempt with twice the recommended dose (20 mg/kg bw) was conducted on day 21 (42 surviving animals) and post-treatment samples were collected on day 35 (35 survivors). However, the egg counts and coproantigen levels increased further (Table 2). Not only among the study animals, but in the whole flock many sheep perished within a few weeks (roughly 300 animals out of originally 1300 ewes and lambs according to the farmer). On day 46, the farmer treated the surviving sheep with closantel (CLOS) in various doses (8.8–20.3 mg/kg bw, same amount of drug (750 mg) independently of the body weight). No rectal samples from day 46 were available, but samples from the 11 surviving sheep of the study population were taken on day 70 (i.e. 24 days post treatment with CLOS). These showed a negative cELISA result and only one of the samples contained *F. hepatica* eggs (16 EPG on day 70 compared to 1223 EPG on day 35). For the surviving 11 sheep, this corresponds to a FECR of 100% (95% CL 99.89–100%) from day 35 to day 70. The FEC development and survival of the animals in the course of the study is visualised in Fig. 3.

**Fig. 4:**
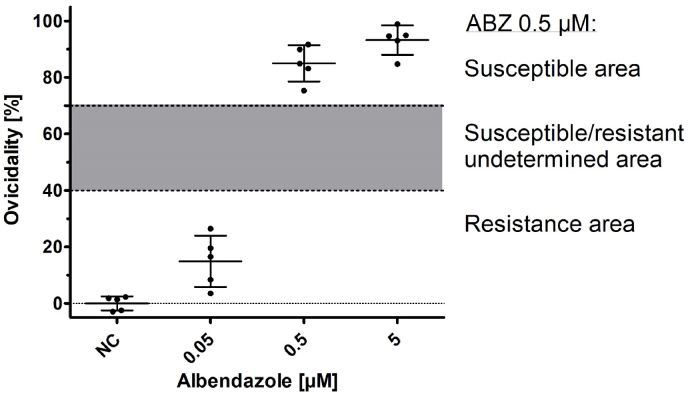
Ovicidal activity (%) of ABZ on *F. hepatica* eggs isolated from faecal samples on farm no. 6: 5 technical replicates per albendazole concentration (0.05; 0.5 and 5 μM) and negative control (NC).

To test the efficacy of ABZ against this phenotypically TCBZ-resistant *F. hepatica*-population, a FMDT was performed using eggs isolated from the pooled sediments after the coprological examination on day 35. The results are shown in Fig. 4. An adequate level of development of the non-treated eggs (negative control) was detected in all replicates (mean: 90.2%) and a high ovicidal activity (85.0%) of ABZ was calculated for the discriminating dose (eggs exposed to an ABZ concentration of 0.5 μM) (Alvarez et al., 2020). Therefore, this isolate can be considered to be ABZ-susceptible according to the FMDT results.

## 4 Discussion

The present study showed seasonal variations regarding the occurrence of *F. hepatica* from 2020 to 2022 on German sheep farms. A significantly higher percentage of coproscopically *F. hepatica*-positive individual animals was found in the winter seasons. *Fasciola hepatica* is a diheteroxenic parasite and a major proportion of its life cycle occurs in the environment including intermediate host stages. Furthermore, the development in the intermediate host is heavily dependent on climate conditions like warm temperatures and moisture. Hence, a seasonal pattern is typical for the epidemiology of *F. hepatica* (Luzón-Peña et al., 1995). The embryonation of *F. hepatica* eggs only occurs when the environmental temperatures exceed 5 °C, and the rate of development increases with higher temperatures (Andrews et al., 2021). In temperate climates of the northern hemisphere, snails are usually infected in spring and summer when the weather conditions are favourable for the miracidia to hatch. After 6–8 weeks, infected snails begin to shed multiple cercariae, which subsequently encyst on plants as metacercariae (Hodgkinson et al., 2018). The infection of the definitive host typically takes place during the end of the grazing season and positive coproscopic results are seen after the prepatent period from the end of autumn onwards and during the winter period.

The number of individual animals infected with Paramphistominae was lower than the number of individual animals positive for *F. hepatica* in the respective season. Interestingly, no significant differences were found between summer and winter seasons regarding the occurrence of Paramphistominae, although the life cycle of the most frequently observed rumen fluke in sheep in Germany, *Calicophoron daubneyi*, is similar to *F. hepatica* with the same intermediate host (Alstedt et al., 2022; Forstmaier et al., 2021). An explanation for that might be, that treatments against rumen flukes are possibly not as frequently conducted in the annual anthelmintic management on many farms compared to treatments against *F. hepatica.* In the routine parasitological monitoring of the sheep and goat health service of the Clinic for Swine and Small Ruminants in Hannover eggs of rumen flukes were not found before 2016 on the farms at the shore of the North Sea (Roden, 2022). In contrast to fasciolosis, infections with rumen flukes in the adult stage are mostly subclinical (Kahl et al., 2021), so that a specific therapy against Paramphistominae was unknown or might not appear necessary for the veterinarians and the farmers and animals remain infected throughout the year. The specific anthelmintic that targets rumen flukes, oxyclozanide, was first licensed in 2019 and most veterinarians only used TCBZ for routine treatment of all flukes. Moreover, we found the occurrence of Paramphistominae limited to the northern part of Germany in our investigations. Most farms, which were coproscopically positive for Paramphistominae in our examinations, are located in the federal States Lower Saxony and Schleswig-Holstein close to the North Sea (Fig. 1). In contrast to that, *F. hepatica*-positive farms were identified throughout the country as shown in the map (Fig. 1). That agrees with a recent study from Alstedt et al. (2022). The authors investigated the geographical distribution of rumen and liver flukes in small ruminants in Germany and found a lower prevalence of patent paramphistomidosis in the southern part of Germany and a higher prevalence in Lower Saxony in the north. In contrast, the prevalence of fasciolosis was higher in the southern federal state Bavaria than in the northern state Lower Saxony. Another recent publication from Forstmaier et al. (2021) investigated the distribution and prevalence of liver and rumen flukes in cattle in Germany. This study also demonstrated a higher rumen fluke prevalence in the north compared to the south while they found the opposite for the occurrence of *F. hepatica*. There are different hypotheses to explain the different distribution of the two trematode groups. In the past, *Paramphistomum cervi* was generally considered to be the predominant rumen fluke species occurring in Europe (Wiedermann et al., 2021). However, recent molecular studies identified *C*. *daubneyi* as the main cause for rumen fluke infections in Germany (Forstmaier et al., 2021; Wiedermann et al., 2021; Alstedt et al., 2022). The same was evidenced in the UK by Gordon et al. (2013), who identified all rumen flukes isolated from sheep and cattle in Scotland as *C*. *daubneyi* by DNA sequencing. In contrast to *P. cervi*, which uses molluscs of the family Planorbidae as intermediate hosts, *C. daubneyi* includes the same intermediate host snail as *F. hepatica* in its life cycle (Kahl et al., 2021). Hence, assuming that *C. daubneyi* is the predominant rumen fluke species in Germany, competitions between liver and rumen flukes might be the reason for the disparate occurrence of these two trematode species, since they compete for *G. truncatula* as an intermediate host (Forstmaier et al., 2021). According to Forstmaier et al. (2021), another reason – especially related to cattle farming – might be the more local livestock breeding system with lesser international animal trading in the southern region leading to a less frequent introduction of rumen flukes into this area. This might also apply for sheep farming. If this is the primary reason, spread of rumen flukes in southern Germany can be expected in the future following the trend to more extensive animal trading.

Generally, the percentage of *F. hepatica*-positive farms was noticeably higher than the percentage of individual animals which were coproscopically positive for *F. hepatica*. On many farms, only individual animals from the flock showed a positive coproscopic result with often very low egg counts. This is noteworthy, since the flocks were specifically selected due to moist pasture conditions or anamnestic *F. hepatica* infections in the past and individual animals in a poor body condition were selected for sampling. The findings of our extensive coproscopic examinations of 1699 individual faecal samples indicate that *F. hepatica* seems to be widely distributed at herd level in German sheep flocks, but in view of the rather low prevalences within the herds, does currently not seem to have a major impact on sheep farming on most farms. One reason for this unexpected finding might be the very dry summers in Germany in the recent years 2018–2020. This might have limited the local habitats where *G. truncatula* snails could live and reproduce and thus, reduced the pressure of *F. hepatica* infection.

Regarding the threshold value for discriminating between a positive and a negative cELISA result, we found a slightly higher level of agreement between FLUKEFINDER ® coproscopy and cELISA, when the cut-off was reduced from 8.0% to 2.0% of the positive control. During the course of the study, we found several individual pre-treatment samples containing at least one *F. hepatica* egg in the coproscopic examination and a concurrent cELISA result clearly below 8.0% of the positive control. The value of 2.0% was the lowest value detected in the cELISA examinations of pre-treatment samples containing at least one *F. hepatica* egg. Therefore, we decided to lower the threshold value according to our personal experience to avoid false-negative cELISA interpretations in low-intensity infections. The Cohen's Kappa increased from 0.797 to 0.805, which both counts as “substantial agreement” according to Landis and Koch (1977), but this difference was not significant since 95% CIs for Cohen's Kappa values were widely overlapping. Palmer et al. (2014) evaluated the sensitivity and specificity of the same commercially available BIO K 201-cELISA kit with a concurrent coproscopic examination of the same samples (using an officially approved sedimentation method using 4 g of faeces for small ruminants and 10 g of faeces for large ruminants and horses and sieving the faecal sample through three sieves of different mesh sizes before sedimentation). The authors also concluded, that the threshold values for the cELISA can be set much lower than the recommendation of the manufacturer without losing specificity, which is in agreement with results in the present study.

Due to the detected low prevalence and egg shedding intensity, only 12 out of 71 examined sheep farms were eligible for the anthelmintic efficacy study. A sufficient efficacy of the respective anthelmintic was observed on most farms (for TCBZ on 10/11 farms and for ABZ on 1/1 farms).

Farm no. 1 was a dairy sheep farm and therefore the use of TCBZ was not allowed. The adulticide ABZ was used as an alternative for this *F. hepatica* infected sheep flock. In fact, the implemented methods to determine anthelmintic efficacy are not optimal to evaluate the efficacy of an adulticide in the field, if the sheep are not kept indoors under fluke-free conditions for at least eight weeks after the last day when infection could have occurred. Adulticides only affect mature flukes, whereas remaining juvenile flukes, if present, may mature within the period of 14 days and influence the outcome of the FECRT and CRT, resulting in misleading results. This problem has already been addressed by Novobilský et al. (2016). In this study, one out of the four initially infected animals was still positive for *F. hepatica* using coproscopy and cELISA on day 14 after ABZ treatment but all animals were negative on day 21. This indicates that delayed clearance of eggs and antigen from the gall bladder caused the positive result on day 14 but was apparently not the result of ABZ-unaffected juvenile flukes that matured into adults after treatment. Fairweather (2011a) defined successful flukicidal treatment as the absence of coproantigens in faecal samples on day 14 post treatment. However, Flanagan et al. (2011b) also observed one individual animal in their study, which had tested negative at earlier sampling-points before, showing a positive cELISA result on day 14 post treatment. They explained positive cELISA values on day 14 post treatment as a result of continued coproantigen release from disintegrating flukes. Similarly, in the present study there was a single sheep positive in the cELISA on day 21 post TCBZ treatment that was negative on day 28, which can be explained by the same phenomenon. Continued coproantigen release might have also occurred in one individual animal on farm no. 2, which still showed a slightly elevated cELISA result on day 15 and a negative coproscopic result on days 15 and 28.

On one of the other potentially TCBZ-treated farms (farm no. 4), the sheep with the highest pre-treatment egg count (154 EPG) and one of the highest cELISA values of the flock (25.04% relative OD value) on day 0 still demonstrated a large number of *F. hepatica* eggs in the post-treatment sample on day 14 post treatment (81 EPG) with a negative cELISA result (0.32% relative OD value) at the same time, leading to a FECR of 83.42% at herd level. The farmer was asked to submit another faecal sample of that respective animal from day 21, but due to unknown reasons, he did not comply with the request. Hence, no further sample of that individual sheep was analysed to determine the egg shedding three weeks after treatment. Since the cELISA result of this sample was clearly negative, it is highly probable, that this individual sheep was still shedding parasite eggs that had been stored in the gallbladder until day 14 post treatment, despite successful flukicidal treatment. Using flukicidal treatment of experimentally infected rabbits, Chowaniec and Darski (1970) concluded that *F. hepatica* eggs were shed in the faeces for up to 35 days after treatment. This means that the chance for false-positive FEC results on day 14 post treatment is high and remaining eggs stored in the gallbladder despite efficient flukicidal treatment may influence the outcome of a FECRT. Exclusively based on the FECRT result, treatment failure would have been suspected for this individual animal and the fluke population on this farm considered to be TCBZ resistant. However, taking the cELISA result into account and also the fact that poor treatment efficacy was only observed for a single animal in the flock, it is highly likely that all flukes were eliminated and only the liver fluke eggs were still present in a high number in the bile system. Therefore, the TCBZ treatment on this farm was considered to be effective. This observation highlights the importance of combining two different diagnostic approaches in field studies on the efficacy of flukicides.

In contrast to farm no. 4, TCBZ resistance was clearly demonstrated on farm no. 6. In the entire examined study group from this flock TCBZ treatment clearly failed, even at twice the recommended dose (20 mg/kg bw). To the knowledge of the author's, this is the first documented case showing that even a double dose of TCBZ is ineffective against a *F. hepatica* population in sheep. Despite TCBZ treatment, a high mortality and an increase in faecal egg counts as well as coproantigen levels were observed on day 14. Since the pathological examination of several perished animals revealed massive infections with immature flukes, the use of a different fasciolicide without activity on juvenile flukes was not indicated. Therefore, a second TBCZ treatment with twice the recommended dose (20 mg/kg bw) was administered on day 21. However, faecal egg counts and coproantigen levels continued to rise even further until day 35 and also the clinical signs of acute fasciolosis associated with a high number of daily animal losses persisted, leading to the reasonable suspicion of TCBZ resistance on this farm.

According to Fairweather (2011a), the term “resistance” should be used with caution, since no standardised protocols and tests are available in *F. hepatica* to prove resistance in the field. Field cases with observations like on farm no. 6 should rather be indicated as “treatment failures” unless a controlled clinical trial confirms the resistance or sensitivity status. Moreover, Fairweather (2011a) lists other reasons for a treatment failure apart from resistance: “(…) e.g., incorrect (under-) dosing, faulty drenching equipment, product failure, reduced metabolism as a result of liver damage, inadequate and incorrect diagnostic tests, even variable quality of drug formulations.”

Regarding farm no. 6 in the present study, under-dosing or faulty drenching equipment can be excluded as being the reason for the unsuccessful treatment since each individual sheep was weighed before treatment and was dosed by AK with exactly 10 mg or 20 mg TCBZ/kg bw, respectively. A disposable syringe was used for the oral administration of the individually calculated dose for each animal. Each animal was monitored after the oral administration to ensure the complete swallowing of the anthelmintic.

Failure of the used product due to quality issues can also be excluded, as the treatments of other flocks from other farms using the same batch of the flukicide were successful. The bottle with the anthelmintic used on farm no. 6 was always stored under the recommended conditions indicated by the manufacturer and was used within the expiry date. The second treatment with the double dose of TCBZ (20 mg/kg bw) was performed using a different TCBZ-containing preparation from a different manufacturer and also the same bottle of this product was successfully utilised on other farms of the study leading to a sufficient efficacy. Regarding the diagnostic tests, a combination of FECRT and CRT was declared as appropriate for evaluating the anthelmintic efficacy against *F. hepatica* (Flanagan et al., 2011a; Hanna et al., 2015; Novobilský et al., 2016). Hence, the only possible alternative explanation to TCBZ resistance in this case is a lack of anthelmintic efficacy due to the induced liver damage and reduced hepatic metabolism of the active substance as a consequence. Juvenile flukes migrate through the liver parenchyma for the first weeks post-infection. During that phase, the migrating flukes cause severe mechanical damage to the liver tissue due to the sharp spines of the tegument as well as the secretion of proteolytic enzymes by the flukes, impairing the organ's vital functions and altering the function of drug-metabolising systems in the liver (Rojo-Vázquez et al., 2012). However, the large number of animals that were included on farm no. 6 in the study and the fact that treatment failed in every single animal, exclude that liver damage is a reasonable explanation for the lack of efficacy of TCBZ.

A final assessment of the TCBZ resistance status would require experimental infection using metacercariae obtained from the population suspected to be resistant. Indeed, eggs collected from these sheep after TCBZ treatment have now been used to infect snails and when metacercaria become available, such experimental infection trials are planned for the future.

The most likely explanation of our observations is an over-use of TCBZ in inadequate dosages in the previous anthelmintic treatments conducted by the farmer. According to the farmer's own statement, TCBZ has been used seven times within the two recent years prior to the examinations described herein. In these previous TCBZ treatments, the sheep were not individually weighed to calculate the exact dosage for each animal. The farmer provided the information that all ewes were roughly dosed for 100 kg bw and all lambs were roughly dosed for 40 kg bw. However, the farmer dosed CLOS at the end of the study using exactly the same amount of drug for a wide range of weights resulting in dosages of CLOS ranging from 8.8 to 20.3 mg/kg bw with a recommended dosage of 10 mg/kg bw. The very frequent use and probably also sometimes underdosing of TCBZ has most probably promoted the emergence of a TCBZ-resistant *F. hepatica* population on the pasture of this farm over the years. According to the farmer, *F. hepatica* has been diagnosed on this farm every year in routine faecal examinations, even in remarkably dry years such as 2020. In contrast to many other farms included in this study, this flock seems to be under infection pressure independent of the climatic conditions. This particular pasture, on which the flock has been grazing, is located behind a dyke and is permanently moist, so that the pasture contamination does not naturally decrease in dry years. Additionally, the summer 2021 was marked by exceptionally heavy rainfalls in this part of the country, resulting in optimal environmental conditions for the intermediate host snails. That might have engendered an overproportional reproduction of the snails and consequently an extraordinary high infection pressure with the arisen TCBZ-resistant *F. hepatica* population on the respective pasture leading to the massive infections we observed in this flock. Another aspect that should be taken into consideration is the geographic proximity of this farm to The Netherlands, where TCBZ resistance is already widespread (Rose Vineer et al., 2020). Since – potentially resistant – *F. hepatica* populations can be unwittingly imported in infected livestock, it cannot be excluded that the first introduction of this resistant *F. hepatica* isolate was due to acquisition of infected animals from The Netherlands. The farmer of farm no. 6 did not purchase sheep from abroad according to his own statement, but it remains unknown, whether neighbouring livestock farms might have purchased infected animals from The Netherlands. This also includes cattle farms since the same *F. hepatica* population can infect different definitive host species and move amongst different hosts according to Beesley et al. (2017).

On that basis, there might also be the possibility that migrating wild ruminants from the neighbouring country carried and shed *F. hepatica*, so that this isolate reached this area.

This field case clearly illustrated the consequences of a lacking TCBZ efficacy: Since TCBZ is the only currently available flukicide affecting the mature as well as the immature fluke stages inside the definitive host, there is no alternative anthelmintic, which could have been used against the early, highly pathogenic stages to prevent further damage caused by the migrating juvenile flukes. Importantly, this field case suggests that whatever the mechanism by which fluke become resistant to TCBZ, it is not possible to overcome this resistance using double doses of the drug. In the absence of available drugs active against the highly pathogenic juvenile stages, flukicides targeting only adult worms obviously have a limited effect on animal welfare in the case of acute infection.

In comparison to all other currently available flukicides, CLOS is the compound which exerts its effects the earliest. A study from George et al. (2017) revealed 90.2% efficacy against *F. hepatica* at a dose of 7.5 mg/kg bw 6 weeks after experimental infection. An older review from Fairweather and Boray (1999) indicated 90% efficacy of CLOS against flukes 6–8 weeks post infection, whereas other available adulticides such as ABZ and oxyclozanide only affect flukes at least 12 weeks post infection. Other flukicides like rafoxanide and nitroxinil show efficacies of ≥90% against immature flukes of 6–8 weeks, respectively (Fairweather and Boray, 1999), but they are currently not approved and available in Germany.

Hence, CLOS should be the drug of choice when TCBZ resistance is suspected to treat fasciolosis as early as possible. The effectiveness of CLOS against TCBZ-resistant *F. hepatica* populations has been reported several times before (Coles et al., 2000; Gordon et al., 2012; Hanna et al., 2015).

The *in vitro* FMDT demonstrated a high ovicidal activity of ABZ against the isolated eggs of this *F. hepatica* population, strongly suggesting that ABZ still has a sufficient efficacy *in vivo*. Coles and Stafford (2001) reported a fluke count reduction of 94% after treating lambs infected with a TCBZ resistant *F. hepatica* isolate with ABZ at a dose of 7.5 mg/kg bw, meaning that TCBZ resistance does not inevitably also lead to a cross-resistance against ABZ. Inversely, there are also reports about sufficient efficacy of TCBZ against ABZ-resistant *F. hepatica* populations (Novobilský et al., 2012; Sanabria et al., 2013).

Observations comparable to this case of TCBZ treatment failure in Germany were very recently reported from Argentina (Larroza et al., 2023). The authors identified a TCBZ-resistant *F. hepatica* population highly susceptible to CLOS *in vivo* and to ABZ as determined by a Fasciola Egg Hatch Test *in vitro*, substantiating that TCBZ resistance does not necessarily lead to cross-resistance to other flukicides.

On all other TCBZ-treated farms, treatment with the recommended dose of 10 mg/kg bw led to a sufficient FECR as well as negative results in the cELISA two weeks after treatment. Since the FMDT was only established in the authors's laboratory during the course of the study, capacities for performing the FMDT with all other field isolates were lacking. Therefore, the FMDT was only conducted with the eggs isolated from farm no. 6.

## 5 Conclusion

The anthelmintic efficacy field trial revealed a sufficient efficacy of the respective flukicide on most of the farms (TCBZ on 10/11 farms and ABZ on the only included dairy sheep farm). Overall, flukicidal resistance does not seem to be a widespread problem on German sheep farms at the moment. However, on one farm there was no apparent activity of TCBZ and this was associated with serious clinical progression, leading to massive losses of animals in the study population and the wider sheep flock on this affected farm. Suspected resistance was corroborated by no faecal egg count reduction at the double recommended dose (20 mg/kg bw) of TCBZ. This is highly relevant regarding animal welfare and economic sheep production and spread of flukicide resistance, especially against TCBZ, should be considered as an emerging and serious problem, as TCBZ is currently the only flukicide effective against all stages of the liver fluke.

## Funding

This work was conducted as collaborative research project of the Freie Universität Berlin, the Federal Office of Consumer Protection and Food Safety (BVL) Berlin, the University of Veterinary Medicine Hannover, and the University of Liverpool. This work was financially supported by the Federal Office of Consumer Protection and Food Safety under the reference number 2019000389.

## Declaration of interests

The authors declare the following financial interests/personal relationships which may be considered as potential competing interests:Georg von Samson-Himmelstjerna reports financial support was provided by Federal Office of Consumer Protection and Food Safety Berlin Mitte. Member of the editorial board of Int. J. Parasitol. Drugs Drug Rest. GvSH.
